# Antiphospholipid-Related Chorea

**DOI:** 10.3389/fneur.2012.00150

**Published:** 2012-10-22

**Authors:** Silvio Peluso, Antonella Antenora, Anna De Rosa, Alessandro Roca, Gennaro Maddaluno, Vincenzo Brescia Morra, Giuseppe De Michele

**Affiliations:** ^1^Department of Neurological Sciences, Federico II UniversityNaples, Italy

**Keywords:** antiphospholipid syndrome, antiphospholipid antibody syndrome, Hughes’ syndrome, APS, anticardiolipin antibodies, anti-β2-glycoprotein I antibodies, lupus anticoagulant, chorea

## Abstract

Chorea is a movement disorder which may be associated with immunologic diseases, in particular in the presence of antiphospholipid antibodies (aPL). Choreic movements have been linked to the isolated presence of plasmatic aPL, or to primary, or secondary antiphospholipid syndrome. The highest incidence of aPL-related chorea is detected in children and females. The presentation of chorea is usually subacute and the course monophasic. Choreic movements can be focal, unilateral, or generalized. High plasmatic titers of aPL in a choreic patient can suggest the diagnosis of aPL-related chorea; neuroimaging investigation does not provide much additional diagnostic information. The most relevant target of aPL is β2-glycoprotein I, probably responsible for the thrombotic manifestations of antiphospholipid syndrome. Etiology of the movement disorder is not well understood but a neurotoxic effect of aPL has been hypothesized, leading to impaired basal ganglia cell function and development of neuroinflammation. Patients affected by aPL-related chorea have an increased risk of thrombosis and should receive antiplatelet or anticoagulant treatment.

## Introduction

As defined by Sanger, chorea is “an ongoing random-appearing sequence of one or more discrete involuntary movements or movement fragments. Movements appear random due to variability in timing, duration, direction, or anatomic location. Each movement may have a distinct start and end point, although these may be difficult to identify since movements are often strung together one immediately following or overlapping with another” (Sanger et al., 2010).

Chorea is a well-known but rare manifestation associated with immune system dysfunction and, particularly, with the presence of antiphospholipid antibodies (aPL).

The aPL represent a heterogeneous population of auto-antibodies directed against phospholipid binding proteins, phospholipids, and other proteins. The most relevant target of aPL is β2-glycoprotein I (β2-GPI). Cardiolipin, prothrombin, annexin V, protein C, protein S, and proteins from the kininogen system are less common ligands. Less frequently, aPL are observed to bind to phospholipid groups, such as phosphatidyl-serine, phosphatidyl-ethanolamine, phosophatidyl-inositol (Arvieux et al., 1997). Lupus anticoagulant (LAC) is a subclass of aPL, detected by a prolongation of phospholipid-dependent *in vitro* coagulation assays. High plasmatic titers of aPL are strongly associated with hematologic, obstetric, neurologic, and cutaneous abnormalities.

The first description of aPL dates back to 1906, when these antibodies were recognized for the false positivity in the Wassermann test because of their ability to bind the phospholipids of bovine heart extracts (Wassermann et al., 1906). Only in the early 1980s, aPL were identified for their association with thrombosis (Harris et al., 1983).

Low and non-pathogenic titers of aPL can be detected in 1–5% of healthy people (Petri, 2000), higher levels of aPL are observed in less than 2% of control subjects (Ginsberg et al., 1995). The prevalence increases with advancing age, reaching highest rates in elderly people with coexisting chronic diseases (Petri, 2000).

Genetic and environmental factors affect the appearance of aPL and their clinical expression. A genetic predisposition has been reported by HLA-linked association studies: HLA-DR4, -DR7, -DRw53, and -DQB1*0302 haplotypes have been correlated with aPL occurrence (Sebastiani et al., 2003). Infections or drug exposure can determine the emergence of aPL, usually without clinical manifestations. The hepatitis C virus, human immunodeficiency virus (HIV), human herpes virus, adenovirus, and parvovirus B19 are the most common viral infections related to aPL detection; aPL can be also detected in bacterial diseases, such as leprosy and syphilis (Sène et al., 2008). Procainamide, phenothiazines, quinine, oral contraceptives, and anti-TNF agents are the drugs that may induce generation of aPL (Ramos-Casals et al., 2008; Dlott and Roubey, 2012).

The presence of persistently high plasmatic levels of aPL, mainly anticardiolipin (aCL), anti-β2-GPI, and LAC antibodies, represents the pathogenic basis of antiphospholipid syndrome (APS). APS, also known as antiphospholipid antibody syndrome (APAS) or Hughes’ syndrome, is a systemic autoimmune condition characterized by a hypercoagulable state, responsible for arterial and venous thrombosis, and pregnancy morbidities.

Antiphospholipid syndrome can be defined primary when it elapses in the absence of any underlying autoimmune disorder (PAPS), or secondary when associated with chronic inflammatory conditions (SAPS; Miyakis et al., 2006). The classification retains today only a nosologic role because there is no evidence of clinical differences between these two conditions (Vianna et al., 1994; Cervera et al., 2002).

Systemic lupus erythematosus (SLE) is the most common cause of SAPS (Cervera, 2008). The positivity of aPL in SLE patients varies from 12 to 30% for aCL (Cervera et al., 1993; Merkel et al., 1996) to 15–34% for LAC antibodies (Love and Santoro, 1990; Cervera et al., 1993). Symptoms and signs of APS are present in 50–70% of SLE patients with aPL after a follow-up of 20 years (Alarcon-Segovia et al., 1992; Petri, 2000). On the other hand, up to 30% of SLE patients with aCL do not develop clinical thrombotic events or pregnancy problems over an average follow-up of 7 years (Alarcon-Segovia et al., 1992). Transition from PAPS to SLE-associated APS has been reported (Mujic et al., 1995) but it is a relatively uncommon event (Mackworth-Young, 2006).

Immunologic conditions less frequently associated with aPL are lupus-like syndrome, Sjögren’s syndrome, rheumatoid arthritis, scleroderma, and systemic vasculitis (Ostrowski and Robinson, 2008).

Ischemic stroke, due to arterial thrombosis, represents the most common neurological manifestation and the major cause of morbidity and mortality in APS (Cervera et al., 2009). Several neurological symptoms and movement disorders have been associated with high titers of APL: migraine (20.2%), seizures (7%), multi-infarct dementia (2.5%), chorea (1.3%), acute encephalopathy (1.1%), transient amnesia (0.7%), cerebral venous thrombosis (0.7%), cerebellar ataxia (0.7%), transverse myelopathy (0.4%), hemiballismus (0.3%; Cervera et al., 2009). Isolated reports have concerned parkinsonism-dystonia (Huang et al., 2008), paroxysmal dyskinesias (Engelen and Tijssen, 2005), tics (Seijo-Martinez et al., 2008), and corticobasal degeneration-like syndrome (Morris and Lees, 1999). Sneddon’s syndrome, characterized by ischemic vascular disease and livedo reticularis, has also been associated with aPL (Caldas and de Carvalho, 2011). Chorea represents the most common movement disorder (1.3%; Cervera et al., 2009) and constitutes one of 19 SLE related neuropsychiatric manifestations established by the American College of Rheumatology in 1999 (The American College of Rheumatology, 1999).

In 1941 Zeller first described choreic movements as a clinical presentation of SLE (Zeller, 1941) and, more than 30 years later, Hughes related chorea to the presence of aPL in the first description of APS (Hughes, 1984).

## Pathogenesis

Anti-β2-GPI should be considered responsible for the thrombotic manifestation of APS (Viard et al., 1992). Several sub-populations of anti-β2-GPI, capable of binding different domains of β2-GPI, are detectable (Shoenfeld et al., 2003). Only the antibodies directed against the first domain of the protein are related to vascular disorders occurring in APS (de Laat et al., 2009). High levels of β2-GPI are present in plasma but its role is not well understood (de Groot and Meijers, 2011). β2-GPI plays a protective function through scavenging activity: it helps to remove lipopolysaccharides (Agar et al., 2011), cellular debris, apoptotic microparticles, and oxided LDL from circulation and to neutralize toxic molecules, such as nitric oxide and superoxide radicals (Balasubramanian and Schroit, 1998; Matsuura et al., 2009; Abdel-Monem et al., 2010; Passam et al., 2011). β2-GPI seems also to contribute to hemostasis regulation, interfering with von Willebrand factor (vWF) activity, and reducing platelet aggregation (Hulstein et al., 2007).

**Figure 1:**
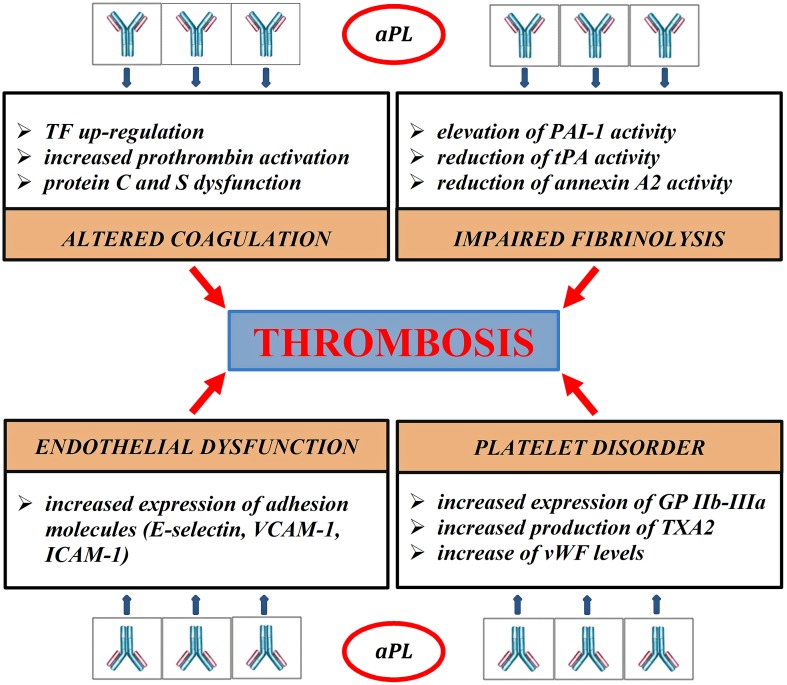
**Pathogenic mechanisms of thrombosis in APS**. aPL disrupt the coagulation balance in many different ways. They bind directly to blood clotting-proteins, such as prothrombin, protein C, protein S, impairing their activity (Malia et al., 1990; Atsumi et al., 1998; Bertolaccini, 2012). aPL reduce the inhibitory effects of protein C on the procoagulant factors Va and VIIIa, inducing an acquired Activated Protein C Resistance (aAPCR). Protein C dysfunction also prolongs Plasminogen Activator Inhibitor-1 (PAI-1) activity, contributing to fibrinolysis disorder (Urbanus and de Laat, 2010). aPL block the tissue Plasminogen activator (tPA) activity directly and inhibit the ability of annexin 2 to potentiate tPA-mediated plasminogen activation (Krone et al., 2010). aPL stimulate the extrinsic pathway of coagulation through up-regulation of tissue factor (TF) mRNA (Lambrianides et al., 2010). APL damage platelet aggregation process. They increase platelet expression of glycoprotein IIb-IIIa (GP IIb-IIIa) and synthesis of thromboxane A2 (TXA2; Robbins et al., 1998; Pierangeli et al., 2008); aPL can also reduce the inhibitory activity of β2-GPI on von Willebrand factor (vWF), promoting platelet aggregation (Hulstein et al., 2007). In experimental models aPL generate a proinflammatory state, increasing the secretion of cytokines, such as IL1 and IL6, and promoting the expression of adhesion molecules, such as E-selectin, intracellular cell adhesion molecules 1 (ICAM1), and vascular adhesion molecules 1 (VCAM1; Pierangeli et al., 2008).

Thrombosis represents the final result of different pathogenic pathways; clot formation, coagulation cascade, endothelial function, and fibrinolysis are variously impaired in APS (Figure 1). The role of aPL in vascular thrombosis has been widely demonstrated; in the most explanatory experimental test, aPL extracted from patients affected by APS and passively infused in rats caused vessel occlusion of mesenteric microcirculation (Fischetti et al., 2005). In experimental models, aPL can bind to endothelial cells, monocytes, and platelets and promote transductional activities. Several receptors, such as annexin A2 (Romay-Penabad et al., 2009), toll-like receptor 2 and 4 (TLR2, TLR4; Pierangeli et al., 2007), apolipoprotein E receptor 2 (Romay-Penabad et al., 2011), and different signaling pathways, such as molecular nuclear-factor-kB (NFkB; Montiel-Manzano et al., 2007) and p38 mitogen-activated protein kinase (MAPK; Vega-Ostertag et al., 2005), have been reported in APS pathology.

Impairment of cerebral circulation was the historical hypothesis used to explain chorea and other movement disorders in APS syndrome. According to this theory, aPL can determine occlusion of lenticulo-striate arteries and produce ischemia of basal ganglia.

Three lines of evidence currently are against this explanation. Firstly, neuroimaging studies have rarely demonstrated ischemic lesions of basal ganglia in patients affected by APS. Secondly, clinical benefits of corticosteroids and other drugs capable of suppressing the immune system support a flogistic/autoimmune hypothesis rather than a vascular theory. Finally, a direct effect of aPL on neuronal tissue is progressively emerging.

Neurological involvement in APS could actually be described as a two-phase process. Initially, aPL could bind to brain endothelium causing endothelial dysfunction (Abbott et al., 2003; Soltesz et al., 2008), leading to microthrombosis and blood vessel inflammation. This causes disruption of the blood-brain-barrier, extravasation of neurotoxic cytokines, and serum proteins, including aPL and activated thrombin (Katzav et al., 2010). In the second phase, aPL could produce direct neural damage. The ability of aPL to bind to neural cell surface has been well-demonstrated. In experimental models, anti-β2-GPI antibodies bind to neuron and astrocyte membranes (Caronti et al., 1998). aPL IgG have been seen to interact with surface-antigens of neuronal cell lines with dopaminergic characteristics (Dale et al., 2011). aPL could modulate neuronal activity in patients affected by aPL-related neuropsychiatric syndromes; these antibodies depolarize synaptic rat brain extracts and may have similar effects in human nerve terminals (Chapman et al., 1999). They could particularly interfere with excitatory pathways through NMDA glutamate receptor over-activation, as demonstrated in rat cerebellar granule cells (Riccio et al., 1998). It has already been proved that aPL can bind cerebral white matter. Immunogold electron microscopy techniques have shown that monoclonal phosphatidylserine-reactive antibodies react strongly with myelin, preferentially with the major dense line formed by the cytoplasmic apposition of the oligodendrocyte plasma membrane (Kent et al., 2000).

## Clinical Features

The Euro-Phospholipid Group analyzed the prevalence of the most relevant clinical and immunological features in a total cohort of 1000 APS patients, derived from 13 European countries; this large multicentre study reported a 1.3% prevalence of chorea (Cervera et al., 2009). Chorea represents a typical onset manifestation of APS in childhood (Cervera et al., 2009), whereas it is most often anticipated by thrombotic events or pregnancy morbidity in adulthood.

The main demographic and clinical data of aPL-related chorea come from three studies conducted on large cohorts (Cervera et al., 1997; Orzechowski et al., 2008; Reiner et al., 2011). Table 1 summarizes the most relevant data from these studies. In adulthood aPL-chorea starts early at an average age of 21 years (Cervera et al., 1997; Reiner et al., 2011), but twelve female patients, 10 non-SLE, and two SLE, had a median onset age of chorea of 44 and 33 years, respectively (Orzechowski et al., 2008).

**Table 1:** **Clinical, diagnostic, and radiologic data from studies about aPL-related chorea**.

Reference	Number of patient (M/F)	Mean age at onset (range)	Isolated aPL positivity*; APS	Unilateral; bilateral chorea at onset	Recidivant forms	Ischemic signs of basal ganglia at CT or MR scan
Cervera et al. (1997)	50 (2/48)	21.2 (6–77)	0; 50 (15 PAPS – 35 SAPS)	18; 21 (11 NR)	16/50	5/31
Orzechowski et al. (2008)	18 (6/12)	44^®^	14; 4	6; 12	6/18	1/18
Reiner et al. (2011)	32 (4/28)	20.6 (9–62)	20; 12 (1 PAPS – 11 SAPS)	15; 15 (2 NR)	8/32	3/31

* Also in the context of an immunological disease.

*^®^Median age in 10 non-SLE female patients*.

*PAPS-SAPS, primary-secondary antiphospholipid syndrome; NR, non-reported*.

aPL-related chorea is more common in females, with a male-female ratio from 1:2 to 1:24 (Cervera et al., 1997; Orzechowski et al., 2008). Hormonal factors probably determine a higher incidence; chorea presentation is often triggered in women by pregnancy or estroprogestinic therapy (Cervera et al., 1997; Reiner et al., 2011).

It is impossible to say if choreic movements are more common in the condition of isolated aPL positivity than in primary or secondary APS. In Cervera’s study, 58% of patients suffered from defined SLE, 12% had lupus-like syndrome, and 30% were affected by PAPS (Cervera et al., 1997). Among SLE patients, 1–3% of adults and 9% of children present choreic movements (Meyer and Kahn, 2000; Joseph et al., 2007; Spinosa et al., 2007). Chorea generally starts within the first year after the onset of SLE, and often precedes the clinical diagnosis (Asherson et al., 1987; Baizabal-Carvallo et al., 2011).

Upon the first encounter with a patient, it may be difficult to clinically distinguish choreic movements associated with aPL from other secondary choreas or specific inherited diseases. As in other hyperkinetic diseases, aPL-related chorea is usually worsened by anxiety and psychological stress and subsides during sleep. In the initial phase, patients attempt to disguise chorea by incorporating it into a purposeful activity; as the disease worsens, the patient can appear fidgety and clumsy.

The presentation of chorea is usually described as subacute, with progressive onset within days or weeks (Orzechowski et al., 2008). aPL-related chorea can involve all body parts; the head, as well as upper and lower extremities can be affected by involuntary movements. Choreic disorder can be unilateral or bilateral: unilateral presentation is common and symptoms often start on one side and successively affect the other (Reiner et al., 2012). aPL-related chorea can be segmental, multifocal, or generalized. Among Orzechowski’s cohort, one patient had segmental choreic movements, eight multifocal, and nine generalized (Orzechowski et al., 2008). The severity is usually mild-moderate (Orzechowski et al., 2008). Choreic movements often appear in a single episode and regress spontaneously or through medication. Reiner et al. (2011) calculated the average duration of chorea in their cohort to be 7.44 weeks. Involuntary movements recur in 25–30% of cases and pregnancy commonly favors their recurrence (Cervera et al., 1997; Reiner et al., 2011).

At onset, aPL-related chorea is often associated with other neurological manifestations, as cognitive impairment, ataxia, epilepsy, psychiatric symptoms, migraine, and dystonia (Orzechowski et al., 2008). Chorea has been related to an increased risk of mitral and aortic valvulopathies, which have been reported in 69% of patients with aPL positivity (Reiner et al., 2012).

## Neuroimaging Aspects

Several neuroradiological investigations have confirmed the rarity of the association between aPL-related choreic disorder and ischemic pathology of basal ganglia. The percentage of patients with aPL positivity who showed basal ganglia ischemic lesions at cerebral CT, MRI, or both, varied between 6 and 16% in four different studies (Cervera et al., 1997; Galanaud et al., 2000; Orzechowski et al., 2008; Reiner et al., 2011).

An important contribution to the study of aPL-related chorea comes from positron emission tomography (PET) analysis. Cerebral PET studies have revealed 18-FDG increased uptake in the striatum, contralateral to the side with involuntary movements (Furie et al., 1994; Wu et al., 2007). Increased FDG uptake may delineate a flogistic state of basal ganglia and can be explained by glucose uptake by infiltrated lymphocytes and resident microglial cells.

## Diagnosis

There are currently no diagnostic criteria for aPL-related chorea. The appearance of choreic movements in patients with isolated aPL positivity, PAPS, or SAPS should suggest this condition. Anamnestic data, such as no family history of movement disorders and lack of previous streptococcal infections; a careful clinical assessment with the exclusion of specific systemic, neurologic, and psychiatric symptoms; and laboratoristic and neuroradiologic exams may exclude other causes of chorea. aPL-related chorea is common in children and it is important to exclude other possible causes, such as Sydenham’s chorea, benign hereditary chorea, DNA repair diseases (ataxia-telangiectasia, ataxia with oculomotor apraxia type 1 and 2), Wilson’s disease, pantothenate kinase-associated neurodegeneration (Walker, 2011).

According to the Sydney criteria revision (Miyakis et al., 2006), to make a diagnosis of APS, the patient must present at least one clinical manifestation of vascular thrombosis or unexplained pregnancy morbidity, and elevated levels of aPL in the plasma. Vascular events include episodes of arterial, venous, or small-vessel thrombosis in any tissue or organ with radiological or histopathological confirmation. Pregnancy morbidity includes at least one of the following: three or more consecutive miscarriages before the 10th gestation week; one or more premature births related to eclampsia, severe preeclampsia, or placental insufficiency before 34 weeks; one or more unexplained fetal losses at or beyond the tenth week of gestation (Miyakis et al., 2006).

Antiphospholipid syndrome recent diagnostic criteria do not consider neuropsychiatric manifestations, despite progressive emerging evidence of a direct interaction of aPL on neural tissue. In our opinion, association of chorea alone with aPL positivity and exclusion of all other relevant causes of acquired or genetic chorea, should suggest a diagnosis of possible APS. This condition may be definable also as “pre APS” because it may anticipate the appearance of thrombotic manifestations (Asherson, 2006; Reiner et al., 2011).

Regarding the available serological assays, aCL and β2-GPI, of both IgM and IgG class, are detected by enzyme-linked immunosorbent assay (ELISA), LAC by phospholipid-dependent clotting assay. International standards for LAC positivity include prolongation of aPL-dependent clotting assay, evidence of inhibition demonstrated by mixing studies, evidence of aPL dependence, and a lack of specific inhibition of any coagulation factor (Wisløff et al., 2002). To allow a diagnosis of APS, persistent aPL positivity must be confirmed on two occasions with an interval of at least 12 weeks (Miyakis et al., 2006).

Although antibodies against β2-GPI are considered responsible of APS, anti-β2-GPI assay does not correlate with the clinical manifestation of thrombosis and fetal loss (Urbanus et al., 2009). Anti-β2-GPI consist in a heterogeneous group of antibodies, only a few of which are responsible for tissue damage. When associated with infections or drug exposure, these antibodies may have no pathogenic role (Shoenfeld et al., 2003; Sène et al., 2008; Dlott and Roubey, 2012). As a functional test, LAC is the most powerful predictor of pathology in APS (Galli et al., 2003). This finding is also confirmed in patients affected by aPL-related chorea: the percentage of LAC positivity varies between 84 and 92% of cases (Cervera et al., 1997; Reiner et al., 2011).

## Therapeutic Aspects

From a neurological standpoint, therapeutic targets in patients affected by aPL-related chorea are the primary or secondary prevention of ischemic accidents and the remission of movement disorders.

There have been no clinic trials on the primary prevention of ischemic stroke in APS; the use of antiplatelet therapy may be useful in individuals with persistently high levels of aPL, especially those with other cardiovascular risk factors (Ruffatti et al., 2011). In individuals affected by recurrent arteriothrombotic events, therapeutic management should include long-term warfarin treatment (Pengo et al., 2012).

Many different therapeutic strategies have been developed for the management of movement disorders associated with aPL. Discontinuation of trigger treatments, such as estroprogestinic therapy, and introduction of anticoagulant or antiplatelet agents represent the first therapeutic step toward chorea remission. In the case of failure, several drugs can be used. Traditional neuroleptics, such as haloperidol (0.01–0.05 mg/kg/die), have proven effective on neural circuits responsible for choreic disorder. This treatment should not last more than 4–8 weeks to avoid the risk of irreversible tardive dyskinesia. Atypical neuroleptics and xenazine could represent effective alternatives. Otherwise, the flogistic/autoimmune etiology of the disorder justifies the recourse to drugs which suppress the immune system, especially steroids. The available studies do not suggest the superiority of one drug over another, particularly because two or more drugs have often been associated. Retrospective analysis on 30 patients showed approximately equal efficacy for steroids and neuroleptics when added on to previous therapy (Reiner et al., 2011). In the absence of remission, a multidrug treatment should be started; combination therapy with haloperidol and steroids has often proven effective in cases of monotherapy failure (Cervera et al., 1997). In addition to symptomatic therapy with dopamine antagonists, the European League Against Rheumatism (EULAR) recommends the combination of glucocorticoids and immunosuppressive agents (azathioprine, cyclophosphamide) to control disease activity (Bertsias et al., 2010).

Isolated case reports suggest the use of intravenous immunoglobulins, plasmapheresis, and treatment with monoclonal antibodies (rituximab) as additional therapeutic options for patients who do not respond to conventional drugs (Lazurova et al., 2007; Tsagalis et al., 2010).

## Methods

The Medline database was searched, using the following terms, both alone and in combinations: “antiphospholipid syndrome,” “antiphospholipid antibody syndrome,” “Hughes’ syndrome,” “APS,” “aCL antibodies,” “anti-β2-glycoprotein I antibodies,” “LAC.” The terms were cross-linked with the neurological manifestations of chorea, dyskinesia, hyperkinesias, and involuntary movement. Available articles published in English, French, and German between the years 1980 and 2012 were reviewed. References noted in relevant articles were also revised. Not all publications accessed are addressed in the article.

## Conflict of Interest Statement

The authors declare that the research was conducted in the absence of any commercial or financial relationships that could be construed as a potential conflict of interest.
